# Supplementary material for: Analysing the impact of COVID-19 risk perceptions on route choice behaviour in train networks
Source: PLoS One. 2022 Mar 3;17(3):e0264805. doi: 10.1371/journal.pone.0264805 (PMC8893614; doi:10.1371/journal.pone.0264805)
Supplement: S1 Table — (DOCX) [file pone.0264805.s001.docx]

Supplementary Information

**S1 Table 1. Questions used for COVID-19 related qualitative indicators (in English).**

| **Variable Name** | **Question/Statement** |
| --- | --- |
| Personal health attitude | Overall, I would rate my health condition as:  Very poor (1)–Very good (5) |
| Personal health anxiety | Select the statement that best describes your feelings, over the past six months:  I never worry about my health (1)–I almost always worry about my health (4) |
| Prosociality | I think it is important to do things for the benefit of others and/or society even if it costs me personally  Completely disagree (1)–Completely agree (5) |
| Perceived control | Overall, I believe that I can control or avoid becoming infected by the coronavirus (e.g.,by limiting social contact, washing hands, wearing a face mask, etc.)  Completely disagree (1)–Completely agree (5) |
| Perceived efficacy | The actions that I personally take to prevent the spread of the coronavirus (e.g., by limiting the number of social contacts, washing hands, wearing a face mask, etc.) are effective.  Completely disagree (1)–Completely agree (5) |
| Vulnerability of family/friends | Overall, I believe that people that I care about are at risk of becoming infected and seriously ill due to the coronavirus outbreak.  Completely disagree (1)–Completely agree (5) |
| Trust in government | I trust the government in effectively handling the outbreak  Completely disagree (1)–Completely agree (5) |
| Media exposure | Did you search for information regarding the coronavirus?  No, I prefer to avoid information regarding the coronavirus or the pandemic; No, I have only received information via regular channels (TV, newspapers, etc.); Yes, I have consciously and at my own initiative sought additional information (via the internet or other sources) |
| COVID-19 experience | Do you know someone who had/has the coronavirus?  No; Yes |
