# Supplementary material for: Analysing the impact of COVID-19 risk perceptions on route choice behaviour in train networks
Source: PLoS One. 2022 Mar 3;17(3):e0264805. doi: 10.1371/journal.pone.0264805 (PMC8893614; doi:10.1371/journal.pone.0264805)
Supplement: Attachment — Submitted filename: plosone_response_round1.docx [file pone.0264805.s002.docx]

Dear Dr Jian Wang and the Editorial Board of PLoS One,

We would like to thank you and the two reviewers for their constructive comments and suggestions. In the following, we present responses to individual reviewers’ comments (which we have numbered for reference). After that, we outline all the changes made to meet journal requirements.

Should the editor or the reviewers have any further questions, we will gladly provide further clarification.

Sincerely yours,

Sanmay Shelat, Thijs van de Wiel, Eric Molin, Hans van Lint, Oded Cats

Reviewer #1: This manuscript presents a two-stage stated choice experiment. The first stage quantifies the impact of contextual factors on travellers’ COVID-19 risk perception, and the second stage analyzes the route choice behaviour by evaluating the trade-off between this risk perception and common travel attributes. This manuscript has a clear structure and takes research on the impact of COVID-19 on travel behaviours, especially for those related to public transportation. Some aspects should be improved and a few detailed comments are listed as follows:

Dear Reviewer 1,

Thank you for your comments. Please find our responses to specific comments below. Changes in the manuscript are noted here (highlighted in bold) and highlighted in yellow in the revised version of the manuscript. Please note that we have rearranged the order of some comments for cohesiveness in our responses.

1. I found the methods used in this manuscript are ordinary, that is, linear regression for analyzing the risk perception and multinomial logit models for evaluating the route choice. Researchers have proposed multiple models on correlation analysis and the discrete selection, and used them to make research related to COVID-19. However, this manuscript ignores the summary of existing models and fails to explain why these models are chosen or what irreplaceable advantages do they have over other models.

Thank you for allowing us to explain our perspective. As indicated in the literature review, there have been a large number of correlational studies on COVID-19 and travel behaviour. These studies (e.g., (Dryhurst et al., 2020; Engle et al., 2020; Gerhold, 2020)) have typically either demonstrated relationships between the pandemic and travel behaviour through descriptive statistics, correlation metrics or linear regression models. We note this in section 2 as such:

The vast majority of studies mentioned above analysed COVID-19 risk perceptions on public transport via **descriptive statistics on a few** Likert scale questions, ridership drops, or aggregate mode share. While some have correlated socio-economic attributes with risk perception (e.g., Beck and Hensher (2020), Kim et al. (2017)), only few have assessed the impacts of different risk determinants on the overall risk perception or how travellers trade-off these factors when making choices…

Only a limited number of studies have analysed the impact of risk determinants and safety measures on choice behaviour. These include studies such as, (Cho and Park, 2021), (Shelat et al., 2021), and (Aaditya and Rahul, 2021), who employ some form of multinomial logit model (e.g., within latent classes or with latent variables). We have also noted these examples in the second- and third-last paragraphs of the literature review section.

In the ultimate paragraph of the review, we note the main advantages offered by the hierarchical information integration (HII) approach adopted in our study. These include:

- better alignment with how decisions are made when abstract concepts such as risk or safety are involved
- being able to study a larger number of variables without overloading respondents
- specifically evaluate which factors after risk perception and the willingness to pay to reduce such risk

Previous transportation applications of the HII theory have—almost exclusively—employed linear regression models for the rating experiment(s) and some form of multinomial logit for the choice experiment (e.g., (Bos et al., 2004; Louviere and Timmermans, 1990; Molin et al., 2017)). Following this precedent and having considered alternatives (i.e., ordinal regression and simple multinomial logit), we decided to use a linear regression model followed by a mixed multinomial logit.

2. In Section 3.2.1, some classification levels for observed attributes of COVID-19 risk perception seem unreasonable in the rating-based sub-experiment. Firstly, the name “intelligent lockdown” is misleading and it should be further distinguished from the “moderate lockdown”; Secondly, levels of infection rate are too close between 6 per 1000 and 10 per 1000, even if they were observed from actual peaks. It may be hard for respondents to distinguish these two levels and I prefer to see them as the same level, compared to 0.2 per 1000 and 100 per 1000.

The term ‘intelligent lockdown’ is indeed confusing for those not familiar with the Dutch COVID-19 policies. It is what the Dutch government called the first lockdown in March 2020. We expect the respondents to be familiar with this term because of its widespread use in governmental announcements and news media. The additional restrictions in the ‘intelligent lockdown’ compared to the ‘moderate lockdown’ have been noted in point 5 of section 3.2.1. We have clarified the origin of the term in the manuscript now:

… the ‘moderate lockdown’ (imposed October 2020) in which large-scale events are forbidden and capacity limits have been set; and the so-called **(by the Dutch government and consequently in the news media)** ‘intelligent lockdown’ (imposed March 2020) where, in addition to the previous level, working from home is strongly advised, dining and entertainment venues are closed, and a maximum of three home visitors are allowed.

We expected the absolute infection numbers to have little meaning for respondents until they were linked with significant points in the preceding year. This is why infection rates at peaks—when additional governmental restrictions were typically placed and there was more intense reporting about the virus in the news—were selected. While it is undeniable that the middle levels are closer to one another than the extreme points, we note that we still find a significant parameter for infection rate. When including infection rates as (effect coded) categorical variables, although we don’t see a substantial improvement in model fit, we do find significantly different parameters for different infection rate levels as shown in the table below. We have not included this table in the manuscript for conciseness.

| **Model** |  | Effect coded infection rate | | | (fixing coeff. for infection: 10 to zero) | | |
| --- | --- | --- | --- | --- | --- | --- | --- |
| # Parameters |  |  |  |  |  |  |  |
| Adjusted R^2^ |  |  | 0.338 |  |  | 0.338 |  |
|  |  | **Coeff.** | **Stand. coeff.** | **p-val** | **Coeff.** | **Stand. coeff.** | **p-val** |
|  | Constant | 1.750 |  | < 0.001 | 1.741 |  | < 0.001 |
| **Risk attributes** | On-board crowding | 0.120 | 0.353 | < 0.001 | 0.122 | 0.359 | < 0.000 |
|  | Number of transfers | 0 | 0 | (fixed) | 0 | 0 | (fixed) |
|  | Sanitization | -0.139 | -0.121 | < 0.001 | -0.133 | -0.116 | < 0.001 |
|  | Face mask policy | -0.210 | -0.183 | < 0.001 | -0.204 | -0.178 | < 0.001 |
|  | Moderate lockdown | -0.157 | -0.097 | < 0.001 | -0.138 | -0.085 | < 0.001 |
|  | *Infection: 0.2 (ref.)* | *-0.245* | *-0.154* | *-* | *-0.278* | *-0.173* | *-* |
|  | *Infection: 6* | *-0.163* | *-0.100* | *< 0.001* | *-0.168* | *-0.104* | *< 0.001* |
|  | *Infection: 10* | *-0.059* | *-0.036* | *0.106* | *0* | *0* | *(fixed)* |
|  | *Infection: 100* | *0.467* | *0.290* | *< 0.001* | *0.446* | *0.277* | *< 0.001* |
| **COVID-19 related**  **characteristics** | Perceived efficacy | 0.173 | 0.128 | < 0.001 | 0.172 | 0.127 | < 0.001 |
|  | Perceived control | -0.122 | -0.094 | < 0.001 | -0.122 | -0.093 | < 0.001 |
|  | Personal health anxiety | 0.132 | 0.074 | < 0.001 | 0.131 | 0.073 | < 0.001 |
|  | Vulnerability of close ones | 0.169 | 0.135 | < 0.001 | 0.169 | 0.135 | < 0.001 |
| **Socio-demographic** | Student | -0.087 | -0.036 | 0.030 | -0.087 | -0.036 | 0.029 |

3. In Table 2, I can’t understand why the type of travel cost, travel time, and age is a ratio. The authors need to check if there are mistakes or not. If not, this manuscript is encouraged to give more descriptions in this table or the text.

Thank you for allowing us to clarify. It is not a mistake—by ‘Type’ we meant variable type (i.e., measurement level), that is, ratio, interval, ordinal, categorical. We have renamed the column to ‘**Measurement Level**’ now, which we feel is a clearer term.

The three variables are of type ratio because they have a well-defined zero point.

4. This manuscript mentioned that the two-stage experiment conceptualized COVID-19 related variables as affecting travellers’ perceived risk, in turn, affects their behaviour. I agree with this idea, however, some attributes such as infection rate can represent the level of risk perception. Thus, I expected to see a discussion on the discrete selection model with these attributes as direct input and further evaluate its difference from this two-stage model. Maybe some interesting conclusions can be drawn and the advantages/disadvantages of this two-stage model can be better represented.

Given the sustained impact of the COVID-19 pandemic, one can expect related risk perceptions to affect travel behaviour. Studying this phenomena with the two-stage hierarchical information integration methodology offers two advantages. First, risk perception is explicitly defined, enabling us to disentangle its impact on behaviour from general dislike of individual attributes (e.g., overcrowding). Second, by having two separate experiments, we are able to consider the impacts of a larger number of attributes, which would have been otherwise too burdensome in a single experiment. Since our experiments are designed with this concept in mind, it is impossible to include the risk attributes (e.g., infection rate, crowding) directly into the choice analysis.

However, we do note similarities between the results from our (two-stage) models and those in Shelat et al. (2021) where a (single-stage) latent class choice model is used. For instance, they also find that high on-board crowding and infection rate are strong deterrents to boarding a train; and that younger travellers and those using the train more frequently are less likely to be concerned about infection risk. While we already note similarities with previous studies in the ‘Results’ section, we add the following in the ‘Discussion and conclusions’ section:

… can use the estimated choice model to predict behaviour under detailed pandemic scenarios and assess the benefits of various risk mitigating policies. **Furthermore, we noted several similarities between the results from this two-stage experiment and earlier single-stage studies, in particular [30].**

5. There are some minor problems that should be corrected:

1) Format error at the beginning of Section 4.1, see “Error! Reference source not found.”

2) All figures in the manuscript is blurring and should be replaced with high-quality figures;

3) The sum of distribution of actual age levels in Table 1 should reach 100%

We have corrected point 1—thank you for pointing out the error.

We have upgraded the figures. Please also note that during the preparation of the review files, the resolution of the figures seem to be lowered. High resolution figures have been made available to the journal.

The actual distribution numbers have been rounded off to the nearest whole number which is why the total sums to 99.

Reviewer #2: The title of this paper is “Analysing the impact of COVID-19 risk perceptions on route choice behaviour in train networks”. To my understanding, this paper should focus on studying the impact of COVID-19 risk perceptions on route choice behaviour. However, a large part of this paper is explaining risk perception and the impact of economic policies and so on. The impact of COVID-19 risk perceptions on route choice behaviour is not clear. Authors should add more explanations on route choice research and provide more related results.

Dear Reviewer 2,

Thank you for your comment. Indeed, including the main results regarding the route choice behaviour up front and explicitly would be a good idea.

The hierarchical information integration approach adopted in this paper captures the notion that decision-makers first form perceptions of reality and then use these to make choices. Thus, the first stage of our experiment focusses on modelling COVID-19 related risk perceptions. This is a critical step because in this stage respondents will make use of these perceptions to choose between routes.

While typically travel time and cost are the main factors, we show in the second stage of our experiment that now risk perceptions also play a significant role in route choice behaviour. We now include these observations explicitly in the ‘Abstract’, ‘Results’, and ‘Discussion and conclusion’ sections, respectively:

… We find that on-board crowding and infection rates are the most important factors for risk perception. Amongst personal characteristics, the vulnerability of family and friends has the largest impact—nearly twice that of personal health risk. **The bridging choice experiment reveals a that while values of time have remained similar to pre-pandemic estimates, travellers are significantly more likely to choose routes with less COVID-19 risk (e.g., due to lower crowding)**. Respondents…

Table 4 shows the final mixed logit models for the short- and long-trip route choice experiments. **All main parameters including the opt-out constant have the expected signs, indicating that travellers are likely to choose routes with lower travel time, cost, and perceived infection risk.** Both travel purpose…

… Next, from the choice observations in each bridging experiment version, we estimated mixed logit models with normally distributed random parameters for travel cost and perceived risk. **As anticipated, the choice models revealed that respondents preferred routes not only with lower travel times and cost but also with a lower perceived COVID-19 risk.** The estimated values…

Moreover, the estimated mixed multinomial logit model, shows the trade-offs respondents make between the conventional attributes (travel times and costs) and COVID-19 risk perceptions while accounting for the heterogeneity amongst respondents’ route choice behaviour. The willingness-to-pay values obtained by combining the models from the two stages further provides insights into travellers choice behaviour that are critical for supply planning, demand management, and cost-benefit calculations.

Outline of changes to follow journal style guide:

- Changed text to double spacing
- Changed title and heading font sizes according to style guide
- Changed author information as per style guide
- Changed figure and table captions according to style guide
- Added the ethics statement to section 3.3

… The survey was offered in Dutch and had an expected completion time of 12–15 minutes. **A data management plan for the survey and the related informed consent was approved by the Human Research Ethics Committee of Delft University of Technology.** Respondents were informed that they participated voluntarily, could stop the survey at any time, and that provided information would be used for research and subsequent scientific publication. They were assured that answers were anonymous. A total of 513 responses…

- Changed S1 to S1 Table, removed list of references from supplementary information, and list of supplementary information at the end of the manuscript

**References**

Aaditya, B., Rahul, T.M., 2021. Psychological impacts of COVID-19 pandemic on the mode choice behaviour: A hybrid choice modelling approach. *Transport Policy* 108, 47-58.

Beck, M.J., Hensher, D.A., 2020. Insights into the impact of COVID-19 on household travel and activities in Australia – The early days of easing restrictions. *Transport Policy* 99, 95-119.

Bos, I.D.M., Van der Heijden, R.E.C.M., Molin, E.J.E., Timmermans, H.J.P., 2004. The Choice of Park and Ride Facilities: An Analysis Using a Context-Dependent Hierarchical Choice Experiment. *Environment and Planning A: Economy and Space* 36, 1673–1686-1673–1686.

Cho, S.-H., Park, H.-C., 2021. Exploring the Behaviour Change of Crowding Impedance on Public Transit due to COVID-19 Pandemic: Before and After Comparison. *Transportation Letters*, 1-8.

Dryhurst, S., Schneider, C.R., Kerr, J., Freeman, A.L.J., Recchia, G., van der Bles, A.M., Spiegelhalter, D., van der Linden, S., 2020. Risk perceptions of COVID-19 around the world. *Journal of Risk Research*, 1-13.

Engle, S., Stromme, J., Zhou, A., 2020. Staying at Home: Mobility Effects of COVID-19. <https://dx.doi.org/10.2139/ssrn.3565703>.

Gerhold, L., 2020. COVID-19: Risk Perception and Coping Strategies. 10.31234/osf.io/xmpk4.

Kim, C., Cheon, S.H., Choi, K., Joh, C.-H., Lee, H.-J., 2017. Exposure to fear: Changes in travel behavior during MERS outbreak in Seoul. *KSCE Journal of Civil Engineering* 21, 2888-2895.

Louviere, J., Timmermans, H., 1990. Hierarchical Information Integration Applied to Residential Choice Behavior. 22, 127-144.

Molin, E., Blangé, J., Cats, O., Chorus, C., 2017. Willingness to pay for safety improvements in passenger air travel. *Journal of Air Transport Management* 62, 165-175.

Shelat, S., Cats, O., van Cranenburgh, S., 2021. Avoiding the Crowd: Traveller Behaviour in Public Transport in the Age of COVID-19. <https://arxiv.org/abs/2104.10973v1>.
